# Supplementary figures and images for: Vertical transmission of African-lineage Zika virus through the fetal membranes in a rhesus macaque (Macaca mulatta) model
Source: PLoS Pathog. 2023 Aug 7;19(8):e1011274. doi: 10.1371/journal.ppat.1011274 (PMC10434957; doi:10.1371/journal.ppat.1011274)

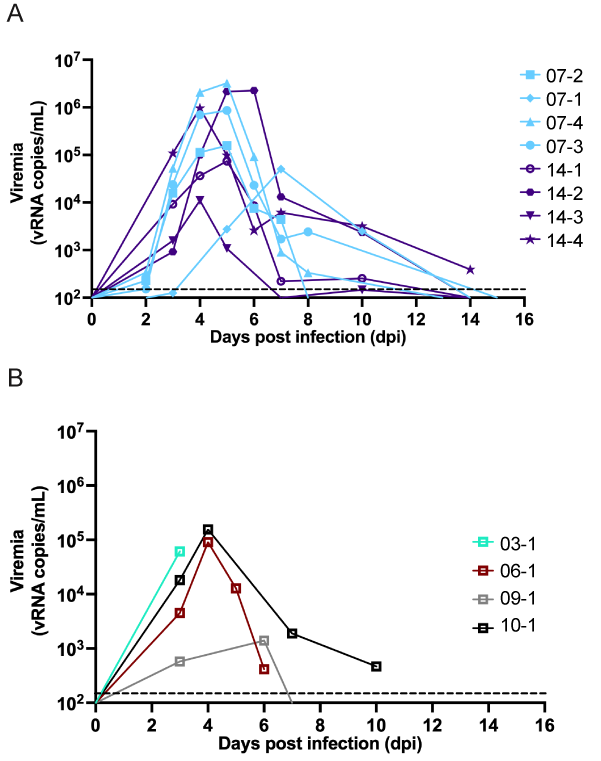

Supplement: S1 Fig — Plasma viremia was determined by RT-qPCR and is represented as vRNA copies/mL from 0 up to 16 dpi. (A) Plasma viremia for the eight animals in the fetectomy cohort. (B) Plasma viremia for the four animals in the perfusion cohort. Only values above the limit of detection (LOD) of 150 copies/mL are shown. The dashed lines represent the LOD of the assay. (PNG) [file ppat.1011274.s001.png]

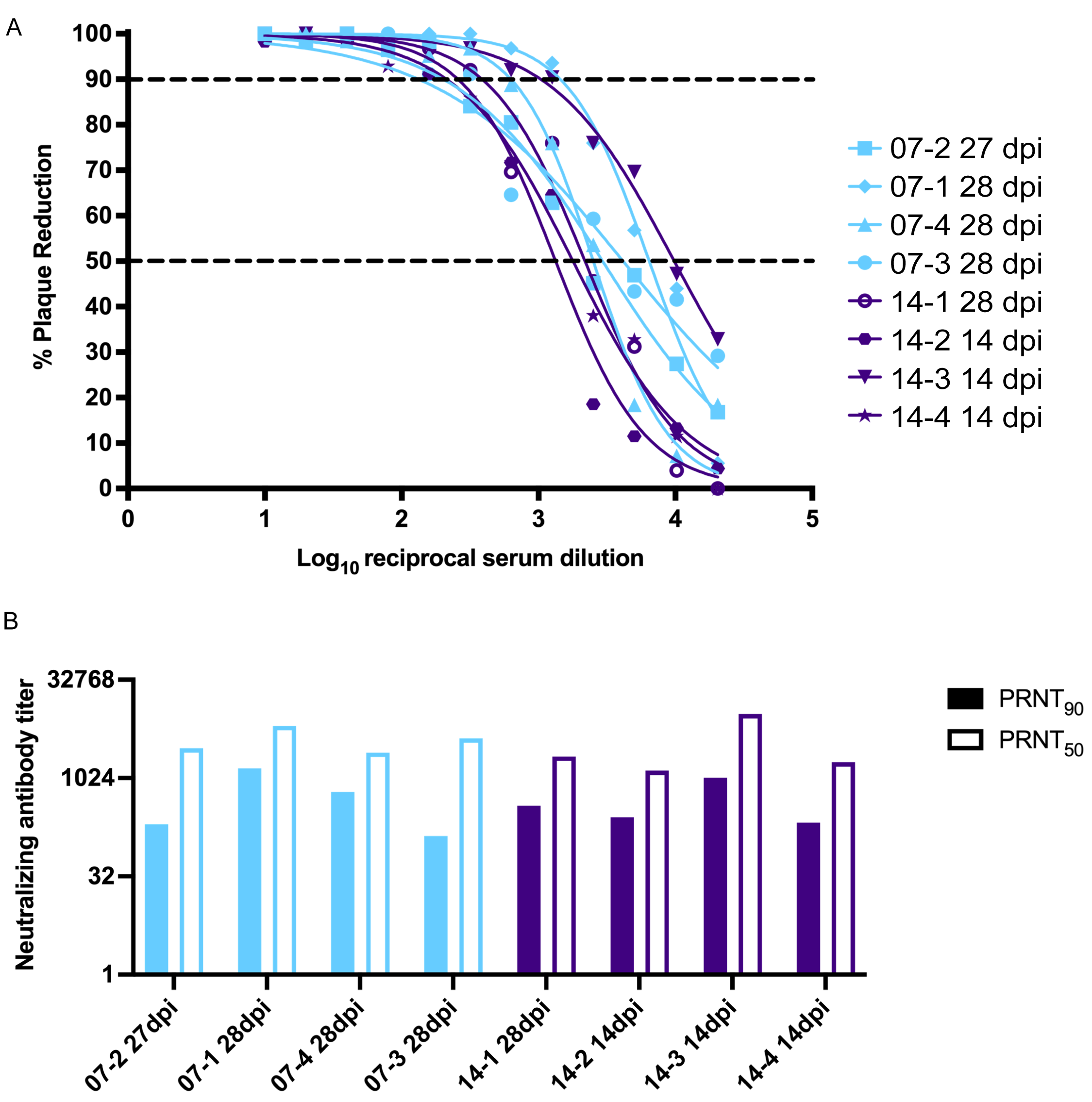

Supplement: S2 Fig — Plaque reduction neutralization tests (PRNT) were performed on serum samples collected prior to infection and at 14 or approximately 28 dpi. Data are expressed relative to infectivity in the absence of serum. A) Neutralization curves. B) PRNT90 and PRNT50 values were estimated using nonlinear regression analysis and are indicated with dotted lines in Panel A. (PNG) [file ppat.1011274.s002.png]

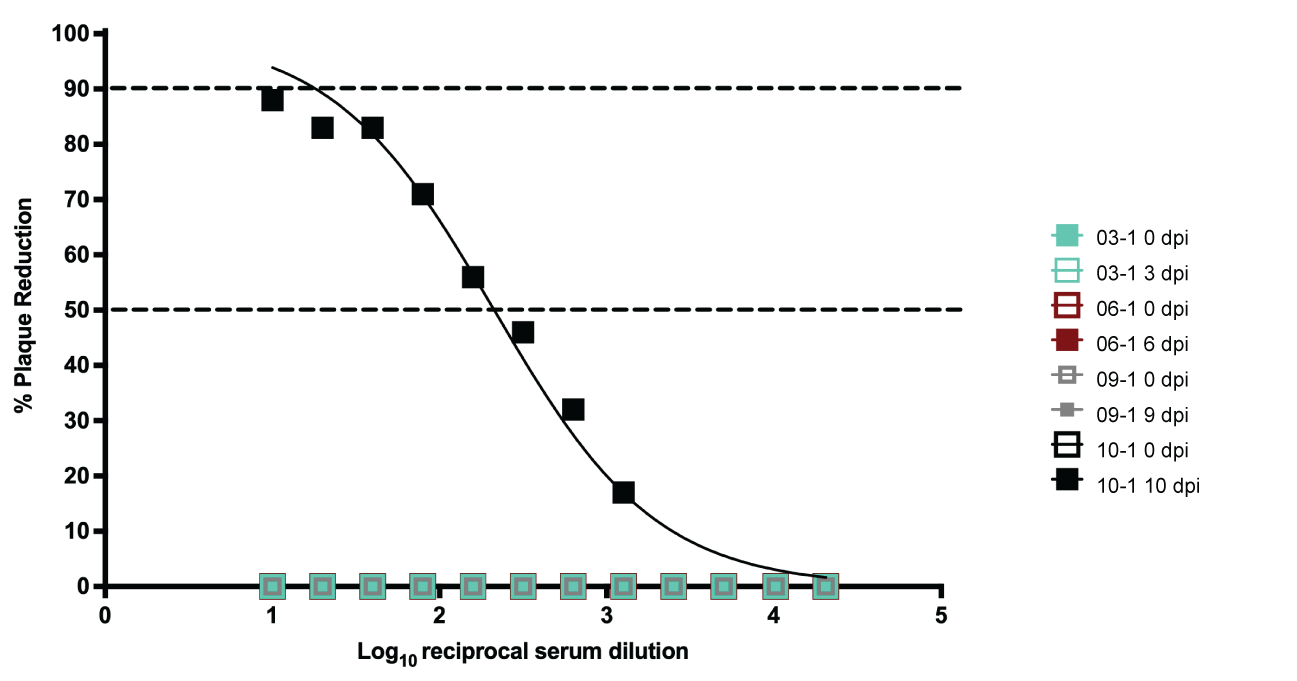

Supplement: S3 Fig — Plaque reduction neutralization tests (PRNT) were performed on serum samples collected prior to infection and on the day of euthanasia. PRNTs are expressed relative to infectivity in the absence of serum. All dams had no neutralizing antibodies prior to inoculation. Symbols for each dam is shown in the right side of the figure, neutralizing antibodies were not detected in any of the dam’s 0 dpi samples. (PNG) [file ppat.1011274.s003.png]

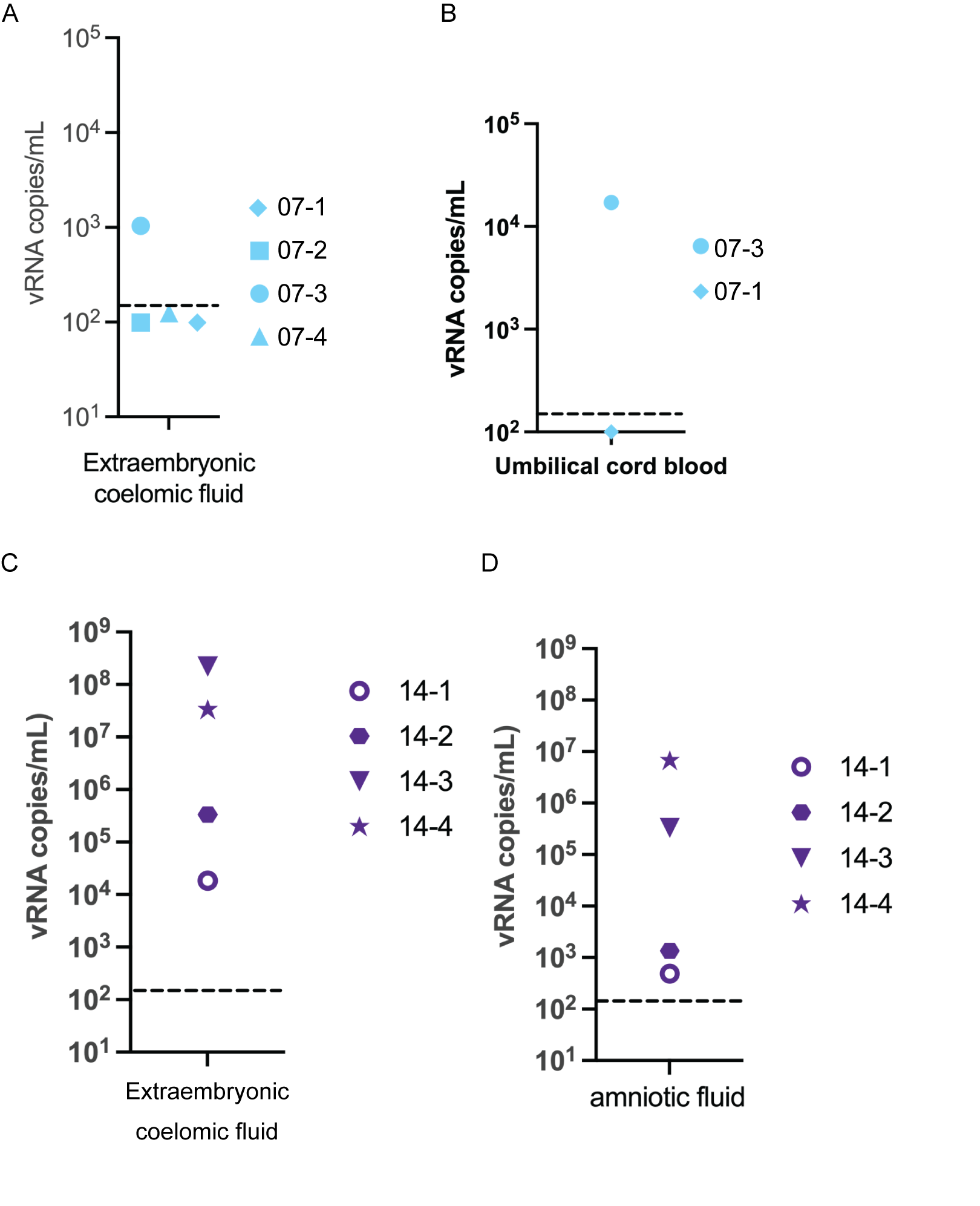

Supplement: S4 Fig — Viral load was determined by RT-qPCR and is represented as vRNA copies/mL. The dashed lines represent the LOD of 150 copies/mL. (A) ZIKV RNA burden in the extraembryonic coelomic fluid at 7 dpi. (B) ZIKV RNA burden in the umbilical cord blood at 7 dpi. (C) ZIKV RNA burden in the extraembryonic coelomic fluid at 14 dpi. (D) ZIKV RNA burden in the amniotic fluid at 14 dpi. (PNG) [file ppat.1011274.s004.png]

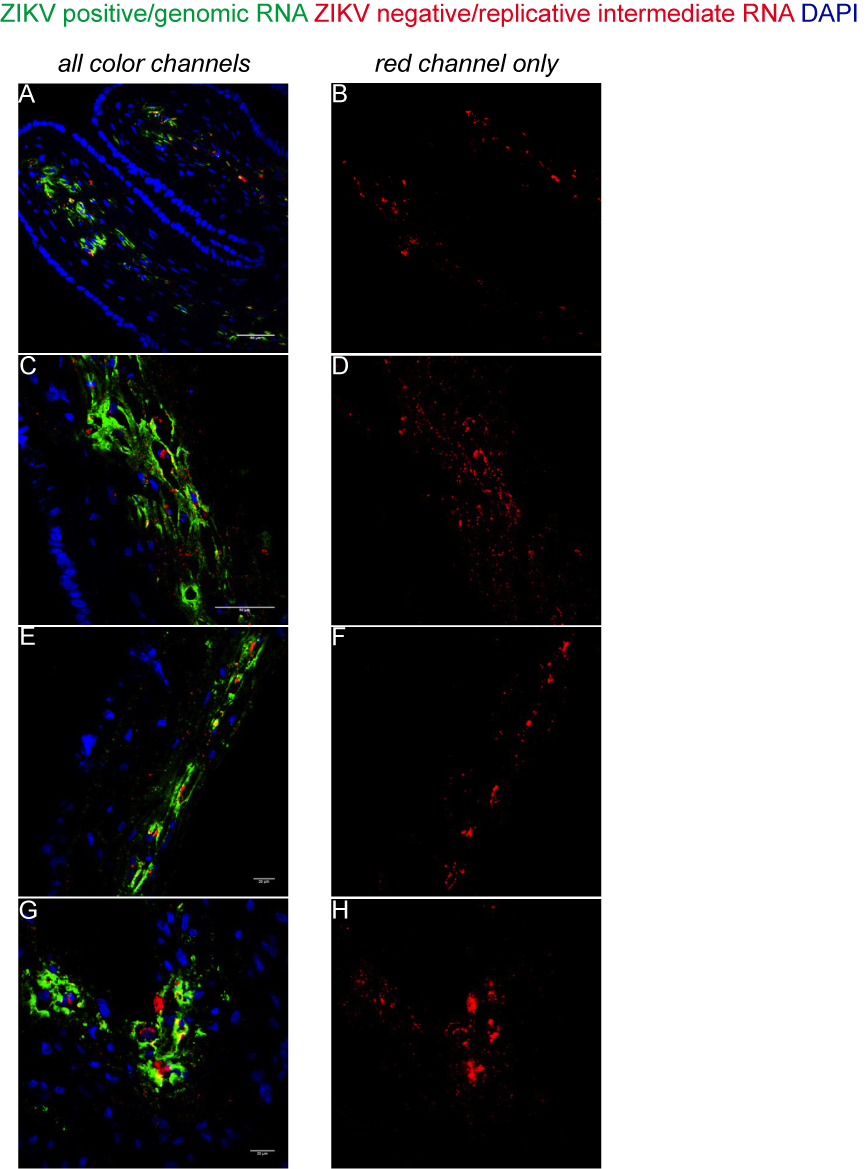

Supplement: S5 Fig — Photomicrographs of multiplex fluorescence in situ hybridization (mFISH) to detect genomic, positive sense ZIKV RNA (green), and replicative intermediate negative sense RNA (red) with nuclear DAPI staining (blue), colocalization of positive and negative sense ZIKV RNA is seen as yellow. (A)(C)(E)(G) panels show merged images of all channels (red, green, and blue). (B)(D)(F)(H) shows only the red channel showing the replicative intermediate RNA detected of the panel immediately to the left. (A)(B)(C)(D) are from case 14–3. (E)(F)(G)(H) are from case 14–4. (A)(C) 50 μm (E) and (G) 20 μm. (PNG) [file ppat.1011274.s005.png]

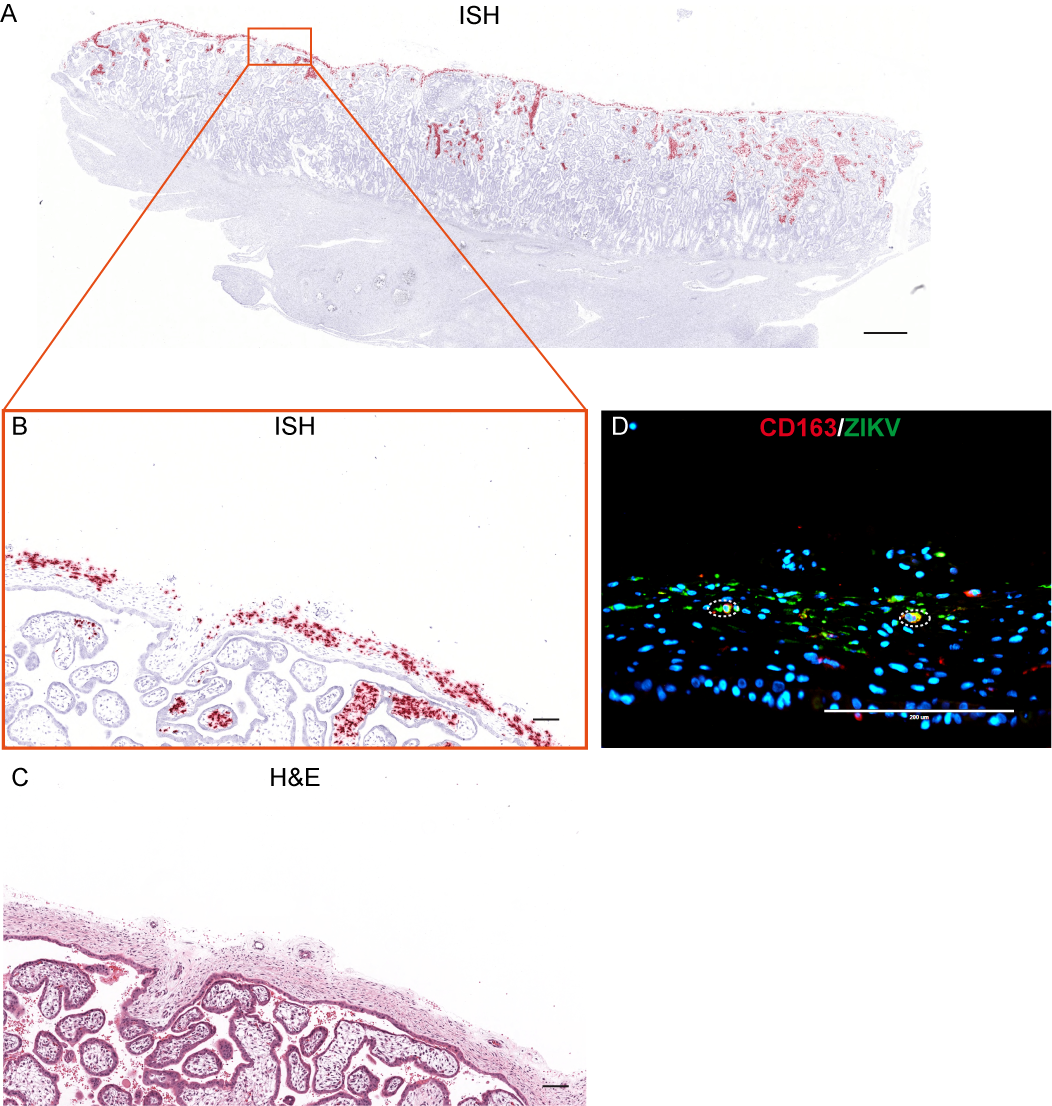

Supplement: S6 Fig — (A) Full thickness decidual/placental tissue section with ISH detection of ZIKV RNA shown as pink staining. The scale bar represents 1000 μm. The Orange square indicates the area magnified in (B). (C) corresponding section H&E stained. (D) Photomicrograph of IF staining for CD163 (red) ZIKV (green) and DAPI (blue) in the chronic plate in a subsequent section of the slide shown in (A) Dashed circle identifies cells that are positive for both CD163 and ZIKV. The scale bar represents 200 μm. (B)(C) Scale bars represent 100 μm. (PNG) [file ppat.1011274.s006.png]

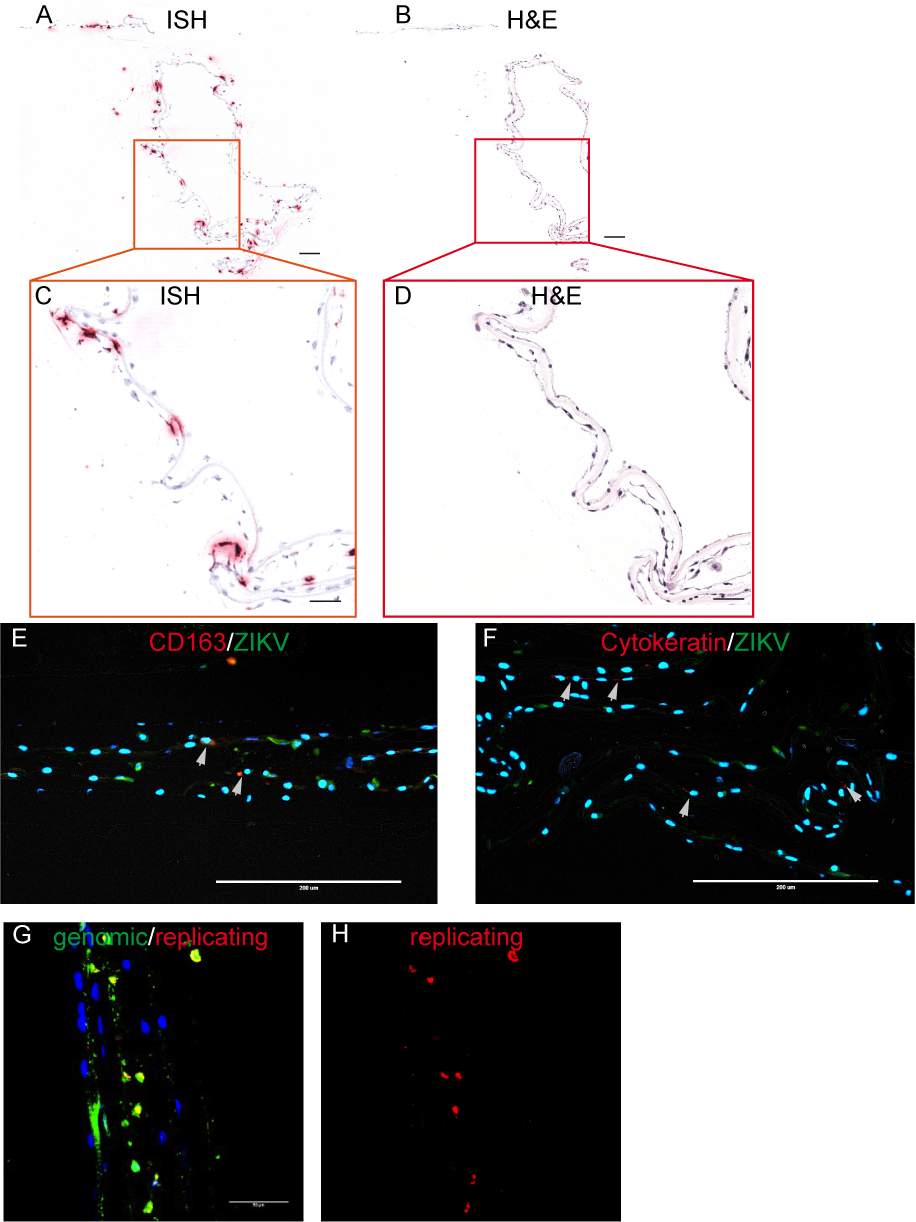

Supplement: S7 Fig — (A) Pink staining indicates ZIKV RNA detected via ISH. Orange square indicates the area magnified in (C). (B) H&E stained section. Red square indicates the area magnified in (D). (A)(B) The scale bars represent 100 μm. (C)(D) The scale bars represent 50 um. (E) IF staining for CD163 (red) ZIKV (green) and DAPI (blue). Little to no colocalization of ZIKV and CD163 was seen as indicated by arrows. (F) IF staining for cytokeratin (red) to identify amniotic epithelial cells, ZIKV (green), and DAPI (blue). Little cytokeratin staining was seen. Positive cytokeratin staining is indicated by arrows and no colocalization of ZIKV and cytokeratin. (E)(F) Scale bars represent 200 μm. (G) Multiplex fluorescence in situ hybridization (mFISH) to detect genomic, positive sense ZIKV RNA (green), and replicative intermediate negative sense RNA (red) with nuclear DAPI staining (blue), colocalization of positive and negative sense ZIKV RNA is seen as yellow. The scale bar represents 50 μm. (H) The red channel of the photomicrograph shown in (G) shows only the replicative intermediate RNA. (PNG) [file ppat.1011274.s007.png]

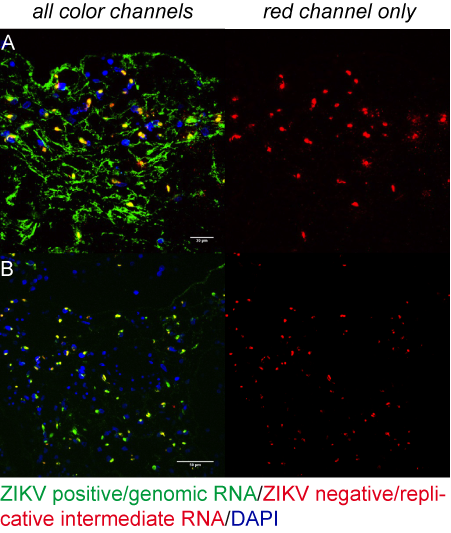

Supplement: S8 Fig — (A)(B) Multiplex fluorescence in situ hybridization (mFISH) to detect genomic, positive sense ZIKV RNA (green), and replicative intermediate negative sense RNA (red) with nuclear DAPI staining (blue), colocalization of positive and negative sense ZIKV RNA is seen as yellow. Image on the left shows all (red, green, and blue) channels and the image on the left shows the red channel (replicative intermediate RNA) alone. (A) The scale bar represents 20 μm. (B) The scale bar represents 50 μm. (PNG) [file ppat.1011274.s008.png]

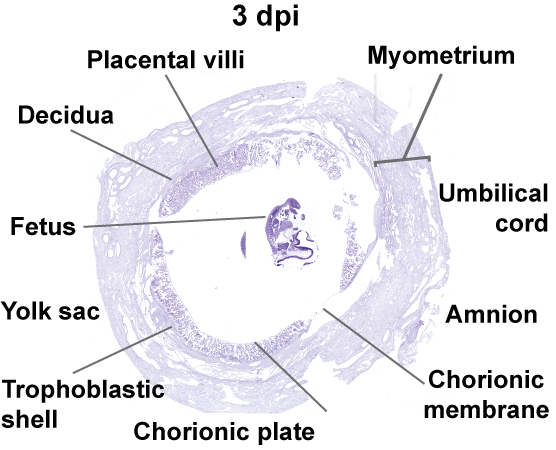

Supplement: S9 Fig — Photomicrograph of a representative coronal section of the gravid uterus evaluated at 3 days post-infection evaluated with ISH. No virus was detected in any of the coronal sections. (PNG) [file ppat.1011274.s009.png]

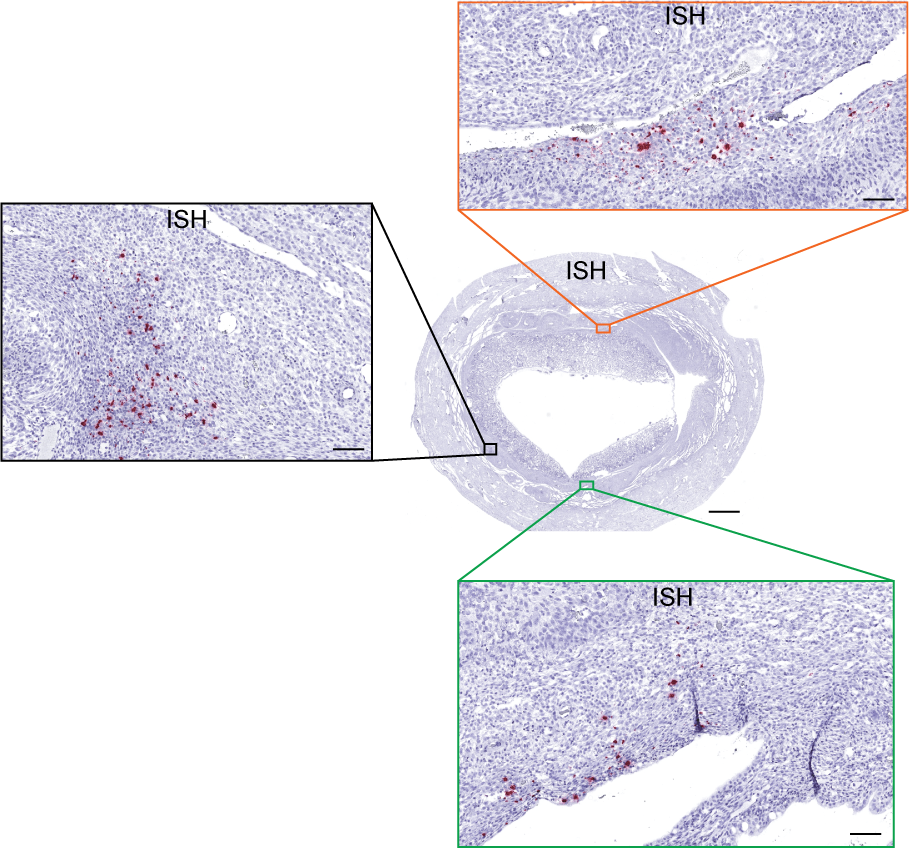

Supplement: S10 Fig — Photomicrographs of ZIKV RNA shown as pink staining detected via ISH in a representative slide from 09–1. Center image shows the full slide. Color boxes indicate areas of the decidua presented at higher magnification. (PNG) [file ppat.1011274.s010.png]

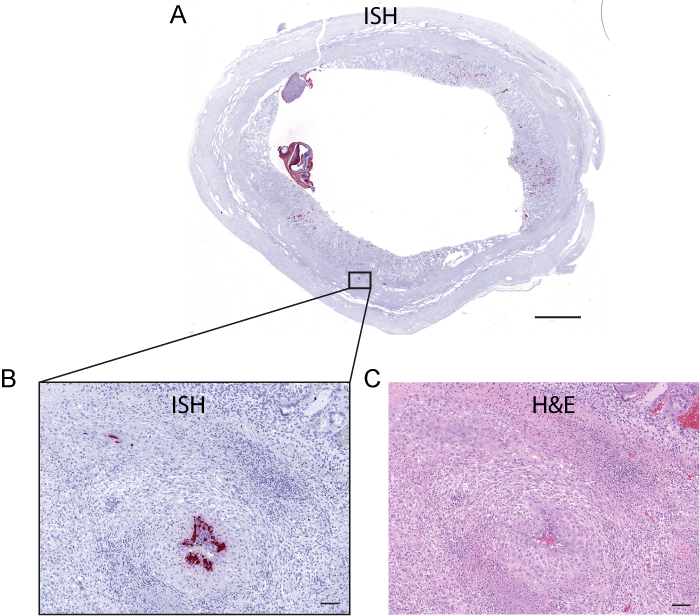

Supplement: S11 Fig — (A) Coronal section of the uterus evaluated for ZIKV RNA via ISH. The scale bar represents 5000μm. The black square indicates the area shown at higher magnification in (B) showing an infected decidual vessel. (C) shows the corresponding H&E staining. (B)(C) The scale bar represents 100 μm. (PNG) [file ppat.1011274.s011.png]

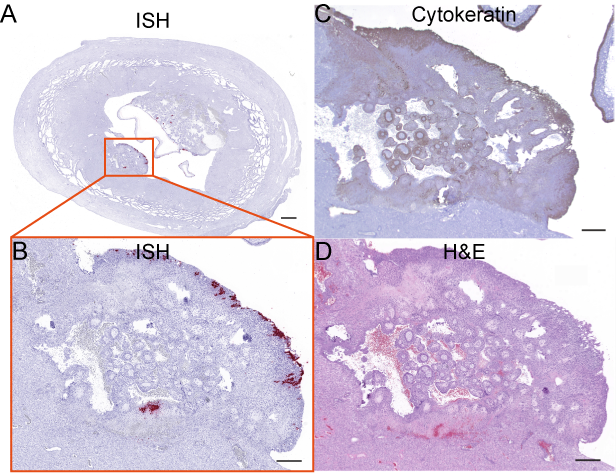

Supplement: S12 Fig — (A) Full slide with pink staining indicating ZIKV RNA detected via ISH. The scale bar represents 2000 μm. The orange square indicates the portion of the slide shown at higher magnification in (B). Corresponding sections of (B) are IHC stained for cytokeratin shown as brown staining in (C) and H&E stained in (D). (B)–(D) the scale bars represent 500 μm. (PNG) [file ppat.1011274.s012.png]

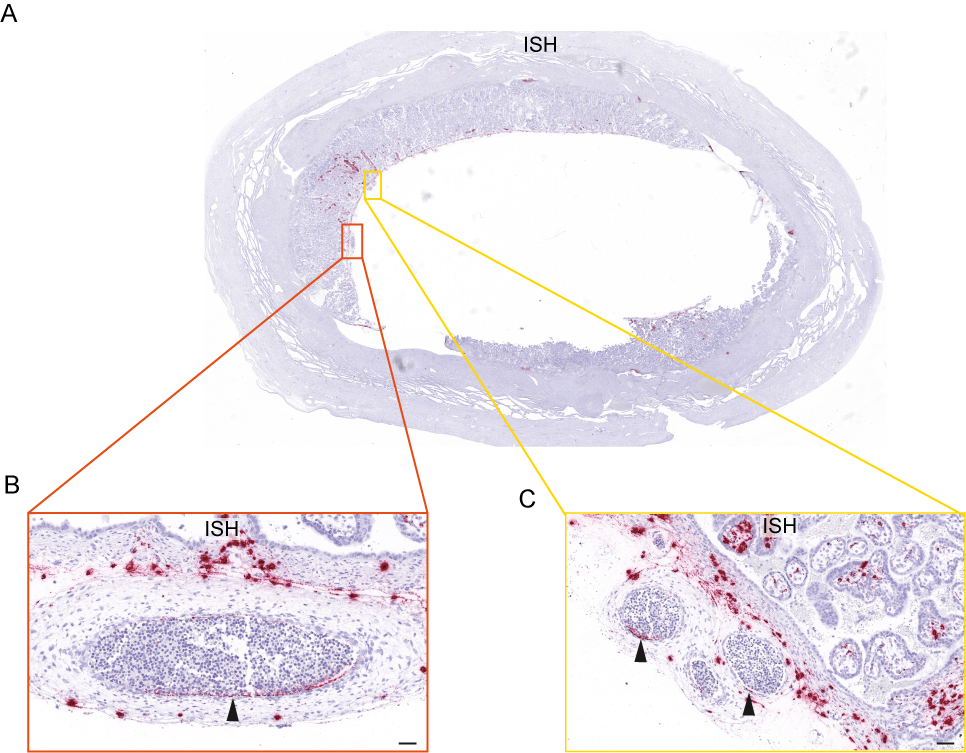

Supplement: S13 Fig — (A) Full slide of ISH detection of ZIKV RNA shown as pink staining. The orange square indicates the portion of the slide shown at higher magnification in (B). The scale bar represents 50 μm. The yellow square indicates the portion of the slide magnified in (C). The scale bar represents 71 μm. Arrows indicated infected endothelial cells. (PNG) [file ppat.1011274.s013.png]

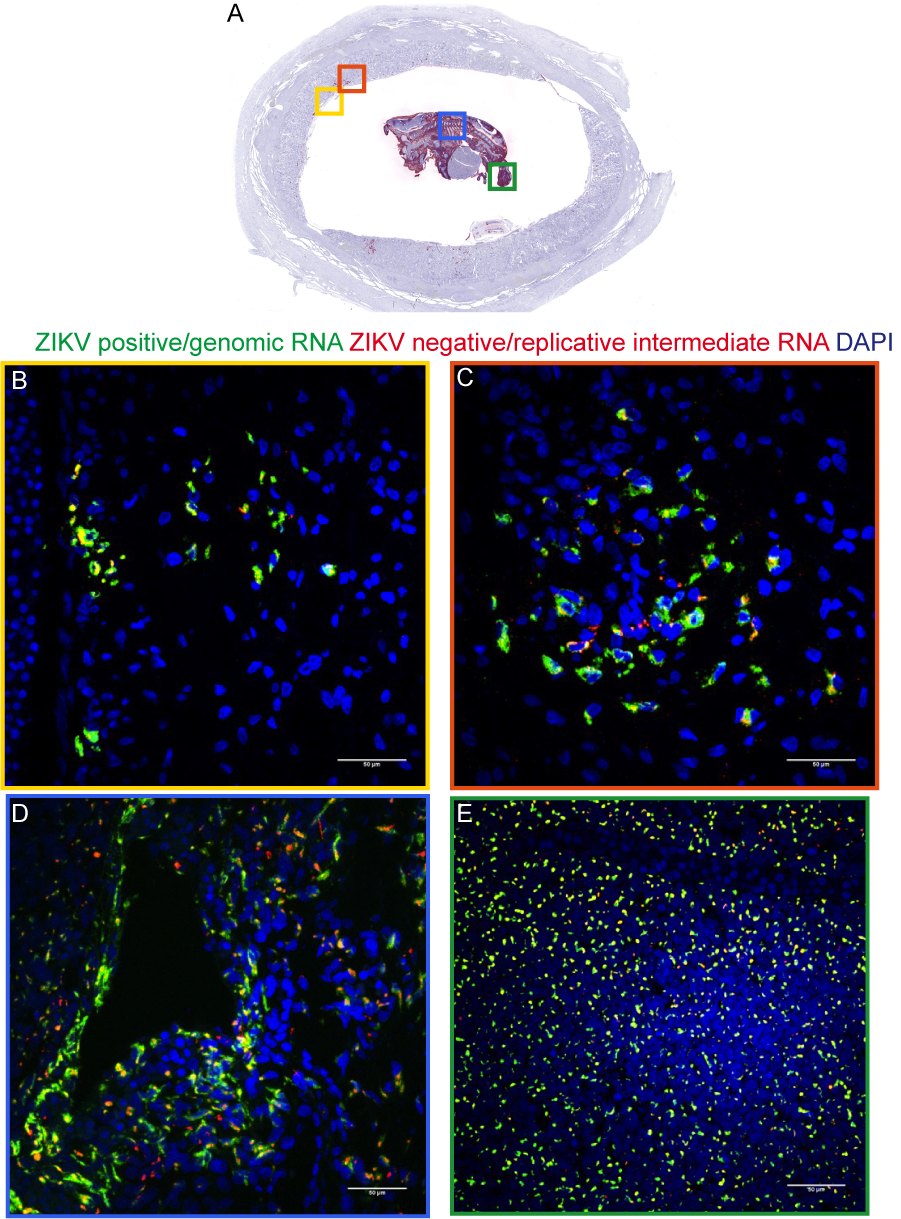

Supplement: S14 Fig — (A) Full slide of ISH detection of ZIKV RNA shown as pink staining. Colored squares indicate areas of the slide shown at higher magnification in subsequent panels. Yellow (B) shows infection near the chorionic plate, red (C) shows infection in the placental villi, blue (D) and green (E) show infection in the fetus. (B)(C)(D)(E) Photomicrographs of multiplex fluorescence in situ hybridization (mFISH) to detect genomic, positive sense ZIKV RNA (green), and replicative intermediate negative sense RNA (red) with nuclear DAPI staining (blue), colocalization of positive and negative sense ZIKV RNA is seen as yellow. The presence of yellow and red staining indicates that there is replicating ZIKV in the chorionic plate, villi, and fetus in this 10 dpi case. The scale bars represent 50 μm. (PNG) [file ppat.1011274.s014.png]

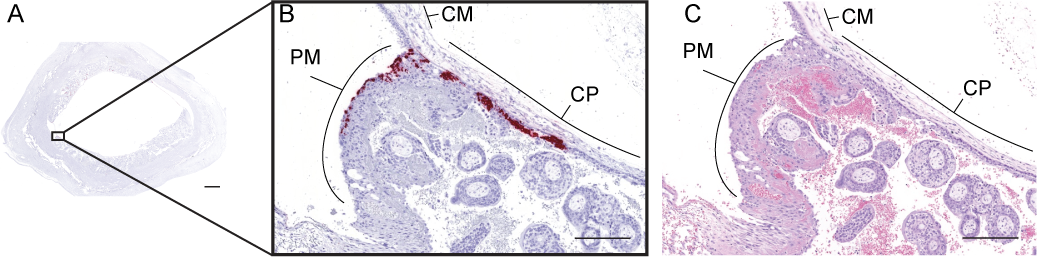

Supplement: S15 Fig — (A) Full slide evaluated with ISH for ZIKV RNA indicated by pink staining. Scale bar represents 3000 μm. Black square indicated the portion of the section magnified in (B) (C) Corresponding H&E stained section. PM = the peripheral margin of the placenta, CM = the chorionic membrane and CP = chorionic plate. The pink staining in the chorionic plate includes trophoblasts of the chorionic plate. (B)(C) Scale bars represent 250 μm. (PNG) [file ppat.1011274.s015.png]

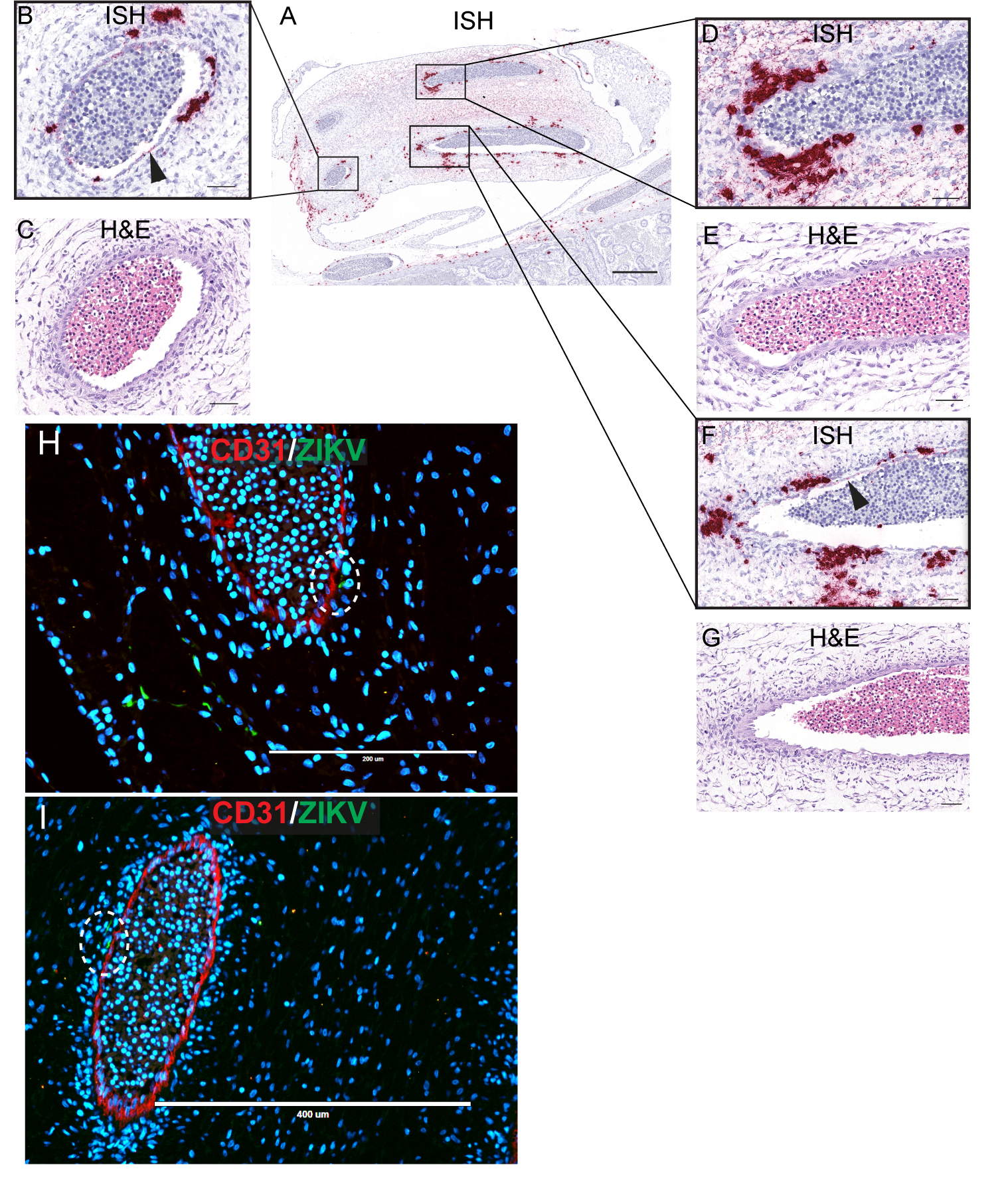

Supplement: S16 Fig — (A) ZIKV RNA detected in the umbilical cord via ISH. Black boxes indicate the areas magnified in (B)(D) and (F). Scale bar represents 250 μm. (C)(E) and (G) show the H&E stained section of the images shown in (B)(D) and (F) respectively. Arrows indicate examples of punctate staining in endothelial cells indicating ZIKV RNA. (B)–(G) Scale bars represent 50 μm. IF staining shown in (H) and (I) for endothelial cell marker CD31 failed to colocalize with ZIKV. Dashed circles indicate areas where ZIKV was identified close to the endothelial cells, but was not colocalized. Scale bars represent 200 μm in (H) and 400 μm in (I) (PNG) [file ppat.1011274.s016.png]

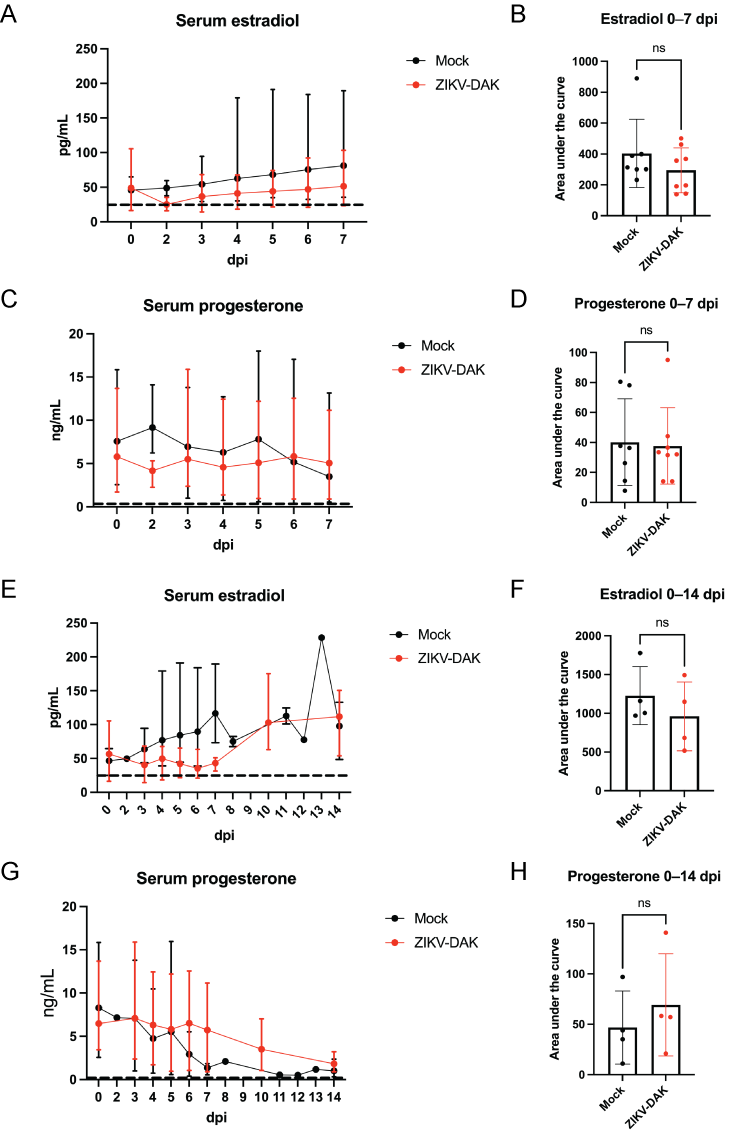

Supplement: S17 Fig — (A)(C)(E)(G) Mean serum levels of estradiol (A)(E) and progesterone (C)(G). Red dots represent mean values for ZIKV-infected dams, black dots represent mean values for mock-infected dams. Ticks represent the range of values for each group. (A)(C) represent the values 0–7 dpi for all eight ZIKV-infected dams from cohort 1 and all eight mock-infected dams. (E)(G) show the values for only the four dams from the 14 dpi group in cohort 1 and their respective controls (n = 4) for serum hormone levels 0–14 dpi. (B)(D)(F)(H) Calculated area under the curve for respective serum hormone levels. Area under the curve of the ZIKV-infected was compared to the mock-infected using Welch’s t-test. There were no significant differences between groups (ns). (B)(D) ZIKV-infected dams (n = 8) are compared to mock-infected dams (n = 7). One dam had two pregnancies randomly assigned to the 7 dpi mock group. Therefore, an average value between her two pregnancies was used in (B) and (C); thus only seven data points are used for the mock-infected dams. (A)(E) Limit of quantification for the serum estradiol assay was 25 pg/mL and is represented by the dashed line. (C)(G) Limit of quantification for the serum progesterone assay was 0.2 ng/mL and is represented by the dashed line. (PNG) [file ppat.1011274.s017.png]

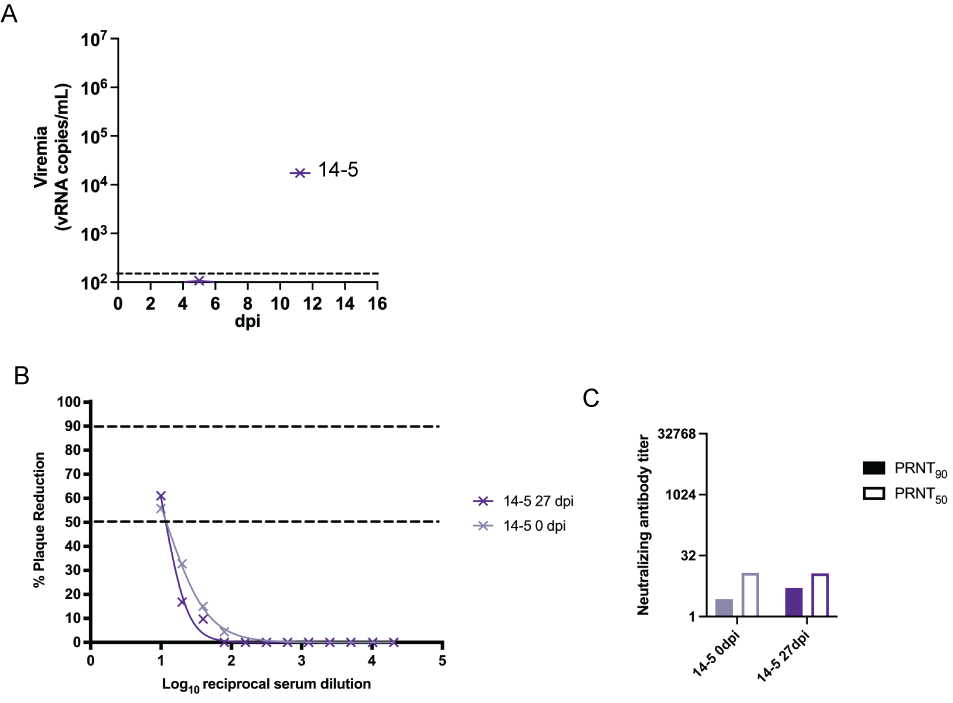

Supplement: S18 Fig — (A) Plasma ZIKV loads were determined by RT-qPCR and are represented as vRNA copies/mL from 0 up to 16 dpi. LOD is represented on the graph with a dashed line. (B)(C) Plaque reduction neutralization tests (PRNT) were performed on serum samples collected prior to infection and 27 dpi. Data are expressed relative to infectivity in the absence of serum. (B) Neutralization curves. (C) PRNT90 and PRNT50 values were estimated using nonlinear regression analysis and are indicated with dotted lines in Panel A. (PNG) [file ppat.1011274.s018.png]

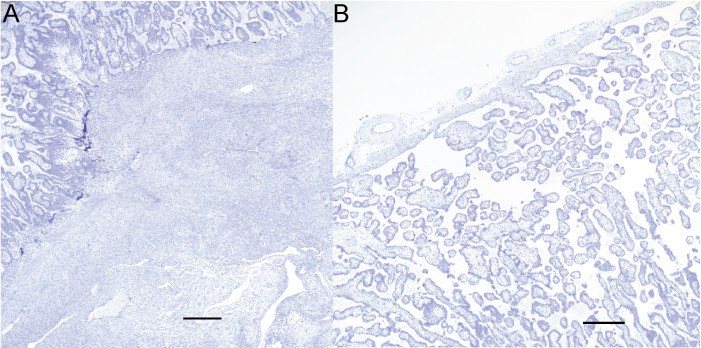

Supplement: S19 Fig — Photomicrographs showing different regions of a placenta from an uninfected control pregnancy. (A) Placental villi with decidua, (B) Chorionic plate with placental villi. (PNG) [file ppat.1011274.s019.png]

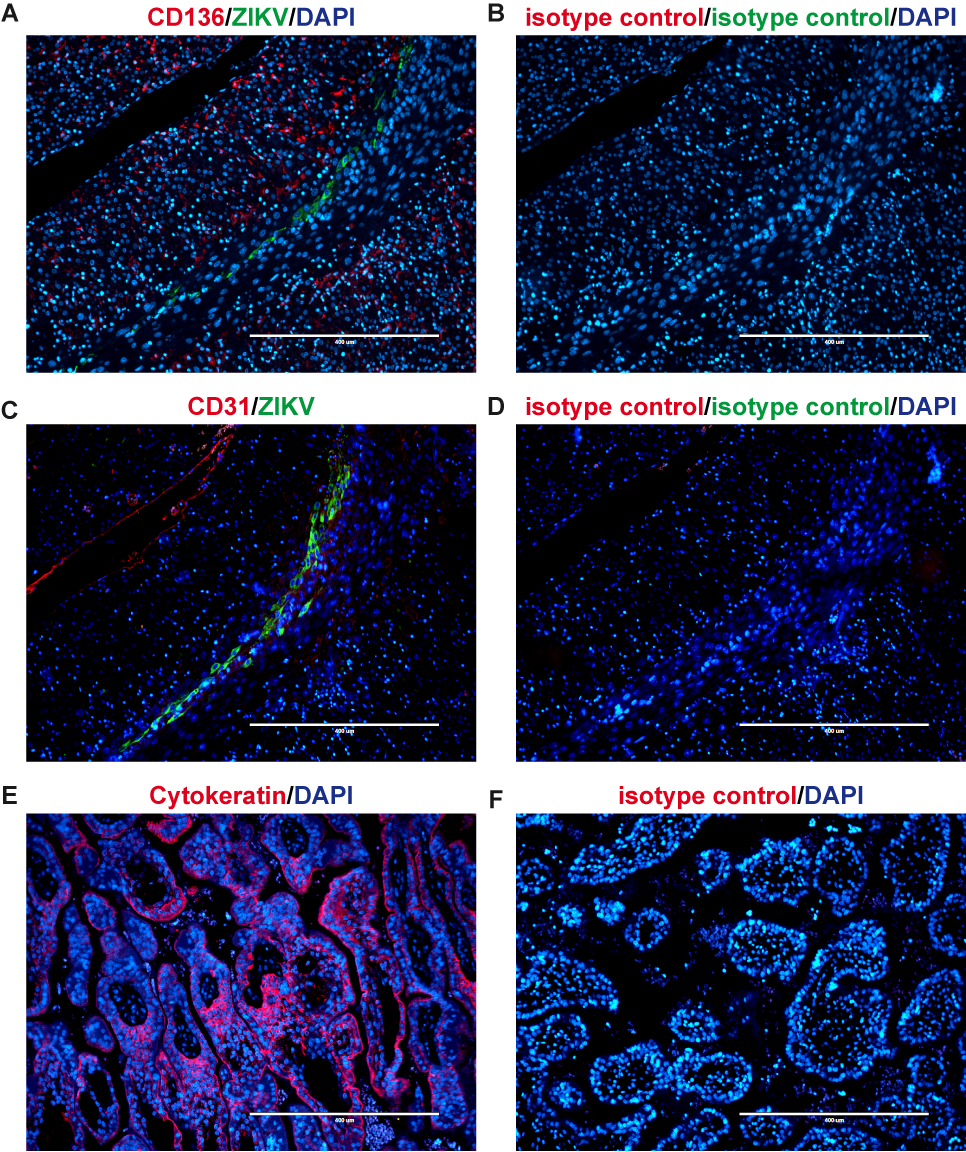

Supplement: S20 Fig — (PNG) [file ppat.1011274.s020.png]
